# Determination of a Measurement Procedure for the Study of Cells’ Dielectric Properties through Descriptive Statistic

**DOI:** 10.3390/bioengineering10080907

**Published:** 2023-07-31

**Authors:** Livio D’Alvia, Barbara Peruzzi, Ludovica Apa, Zaccaria Del Prete, Emanuele Rizzuto

**Affiliations:** 1 Department of Mechanical and Aerospace Engineering, Sapienza University of Rome, 00184 Rome, Italy; ludovica.apa@uniroma1.it (L.A.); zaccaria.delprete@uniroma1.it (Z.D.P.); emanuele.rizzuto@uniroma1.it (E.R.); 2 Bone Physiopathology Research Unit, Bambino Gesù Children’s Hospital, IRCCS, 00146 Rome, Italy; barbara.peruzzi@opbg.net

**Keywords:** descriptive statistic, metrological evaluation, dielectric properties, biosensor, non-invasive measurements, cancer cell lines, osteosarcoma, breast cancer

## Abstract

This paper presents a measurement procedure for analyzing the dielectric properties of cells using descriptive statistics. The study focuses on four cancer cell lines (MDA-MB-231 and MCF-7 breast cancer, SaOS-2, and 143B osteosarcoma) and DMEM culture medium, utilizing the Lorentzian fit model of the return-loss function. The measurements are performed using a circular patch resonator with a 40 mm diameter, powered by a miniVNA operating in the frequency range of 1 MHz to 3 GHz. Eight specimens are prepared for each group to ensure reliability, and the return loss is recorded ten times for each specimen. Various statistical parameters are calculated and evaluated, including the average value, standard deviation, coefficient of variation, and relative error between the average and the first values. The results demonstrate that one single acquisition highly represents the entire set of ten data points, especially for the resonant frequency, with an accuracy error lower than 0.05%. These findings have significant implications for the methodological approach to detecting cells’ dielectric properties, as they substantially reduce time and preserve the specimens without compromising the accuracy of the experimental results.

## 1. Introduction

Over the past two decades, the measurement of dielectric properties has profoundly impacted various scientific fields, including industry, building, heritage materials, and soil pollution assessment [1,2,3,4,5,6,7]. In recent years, microwave-based sensors have emerged as valuable tools in the biological and biomedical domains, providing non-invasive methods for the early-stage prognosis of diseases, including malignancies [8,9,10]. The measurement of the dielectric properties of biological tissues has proven to be highly beneficial in biomedical and healthcare applications due to its high sensitivity, versatility, and non-invasiveness [11,12,13,14].

Nowadays, there are several microwave-based techniques available for measuring the dielectric properties of a huge variety of materials, such as complex impedance measurements at low frequencies using an impedance analyzer [15] or resonance methods at high frequencies [16] using a Vector Network Analyzer (VNA). In this work, we focused on the latter technique since it offers several advantages, such as the possibility to obtain both amplitude and phase information of the signals and measure the scattering parameters. Moreover, working at high frequencies offers several advantages when measuring the dielectric properties of cells, like increased sensitivity to subtle changes and a reduced influence of electrode polarization that occurs when charges build up at the interface between the cell and the measurement electrodes (i.e., required by the impedance analyzer) [17,18,19]. Indeed, this methodology enables the analysis of living tissue properties through non-invasive measurements of scattering parameters or complex permittivity [20,21,22], allowing the identification of pathological conditions based on variations in tissue dielectric properties [23]. For instance, Hardinata et al. [24] proposed a complementary split-ring resonator and tested it on animal tissue samples such as chicken, beef, pork, and veal. Similarly, Deshours et al. [25] described the use of a microstrip ring resonator with coplanar access for the dielectric characterization of biological tissues. Microwave probes have also been utilized to characterize cancer tissues and cells, yielding significant results. Hussein et al. [26] evaluated the dielectric properties (dielectric loss, dielectric constant, and conductivity) of a healthy non-tumorigenic cell line (MCF-10A), four breast cancer cell lines (Hs578T, MDA-MB-231, MCF-7, and T47D), and one colon cancer cell line (HT-29) using an open-ended coaxial probe in the 0.2–13.6 GHz range. Macit et al. and Odelstad et al. [27,28] used an open-ended coaxial probe to measure the dielectric property level of human fetal osteoblastic (hFOB) and osteosarcoma (SaOS-2) cell lines, respectively, in the 0.5–10.0 GHz and 2–50 GHz ranges. Oueslati et al. [29] designed a metamaterial split-ring resonator to analyze colorectal (SW620) and breast cancer (Hs578T and MCF-7) cell lines in the 1.0–8.0 GHz range, effectively distinguishing between cells based on different resonance frequencies. Furthermore, our previous works [30,31] employed a circular patch resonator to detect differences among four cancer cell lines (SaOS-2, 143B, MCF-7, and MDA-MB-231) and their aggressiveness. We analyzed the return-loss signals using the Lorentzian fit model, investigating differences in three main parameters of interest: minimum return loss (*minRL*), resonance frequency (*fr*), and full width at half maximum (*FWHM*) within the 1–3 GHz range. The purpose of the sensor described in [31] is to assess the dielectric properties of cells and biological samples in a non-contact and non-invasive manner. Unlike open-end methods that require probe immersion and risk potential sample contamination, the patch sensor proposed here is placed under the culture plate, ensuring the contactless evaluation of the dielectric properties. This design feature enhances the reliability and integrity of the measurements, making it an optimal choice for studying biological samples.

It is worth noting that all these studies lack a standardized testing methodology for evaluating cells’ dielectric properties, and the results are sometimes obtained by averaging multiple acquisitions or based on a single acquisition. Additionally, to the best of the authors’ knowledge, the validity of the methodology has yet to be evaluated in statistical and metrological terms through repeated measurements of the samples.

Therefore, this paper proposes a comprehensive descriptive statistical analysis of a dataset comprising 400 return-loss measurements, for a total of 1200 data points, to establish an optimal measurement procedure for microwave-based sensors. The subsequent sections are structured as follows: Section 2 provides an overview of the cell lines used, the experimental setup, and the experimental process, including the procedure and statistical analysis. Section 3 presents and discusses the obtained results, while Section 4 summarizes the conclusions and significant achievements.

## 2. Materials and Methods

### 2.1. Cell Line and Sample Preparation

The sensor we devised in [31] was developed to distinguish between cell lines originating from different cancer types and with different levels of aggressiveness. In view of this, we focused our attention on two human breast adenocarcinoma cell lines—MCF-7 and MDA-MB-231 [32,33]—and two pediatric human osteosarcoma cell lines—SaOS-2 and 143B [34,35,36,37]—to assess their dielectric response and included the pure culture medium DMEM for control baseline assessment [38]. Notably, SaOS-2 and MCF-7 represent low-aggressiveness osteoblast-like osteosarcoma and low-aggressiveness breast cancer cell lines, respectively, while 143B and MDA-MB-231 correspond to high-aggressiveness lung-tropic metastatic osteosarcoma and high-aggressiveness bone-tropic breast cancer cell lines [39,40].

To conduct the experiments, the cells were seeded in standard 60 mm Petri dishes, considering their growth rate to ensure a similar number of cells at the time of the experiment, which took place 24 h later. The dishes were then placed in an incubator at 37 °C with 5% CO_2._ All cell types and the pure DMEM culture medium were maintained in 1.5 mL of DMEM throughout the measurements. Eight replicates were prepared for each biological sample, and an equivalent number of replicates was prepared for the culture medium (DMEM) as a control group to establish a baseline for comparison. Ten subsequent acquisitions were performed for each sample to determine if one acquisition could represent the average value across multiple measurements. Therefore, 400 return-loss (RL) signals were collected for further descriptive statistical analysis with the experimental system described below.

### 2.2. Experimental Setup

**Figure 1:**
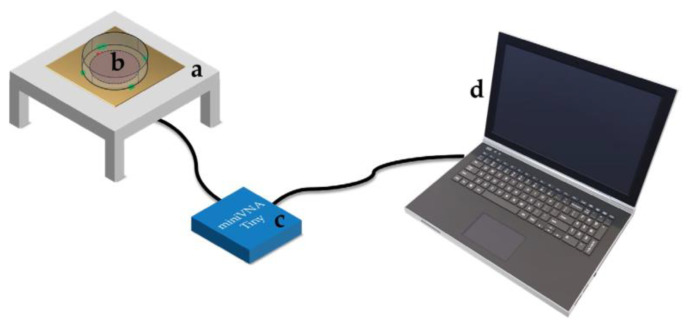
Experimental setup based on (**a**) sensors, (**b**) Petri dish in which cells or pure medium are placed, (**c**) the MiniVNA, and (**d**) a notebook for data acquisition.

Figure 1 reports the experimental setup. In particular, the proposed probe comprises a square dielectric grounded layer (L = 100 mm) and a circular patch resonator with a 20.00 mm radius. A SubMiniature ver. A (SMA) connector was placed on the conductive edge and was employed to measure the dielectric properties of the cell line samples. The probe was realized with Rogers *RO4835* laminate with a dielectric layer of 760 µm and two external layers in copper with a thickness of 35 µm. *RO4835* shows a dielectric constant 𝜀*_RO4835_* and a loss tangent tan*_RO4835_*(𝛿) of 3.66 and 0.0031, respectively. The operating frequency was computed through the following Equation (1) [41]:

(1) $$f_{r}=\frac{c\cdot \gamma_{1,1}}{2\pi r_{e}\sqrt{\varepsilon_{e}}},$$

with *γ*_1,1_ as first zero of the derivative Bessel function of order one (*γ*_1,1_ = 1.84), *r_e_* as the effective radius, and *ε_e_* as the effective dielectric permittivity. The probe was initially designed and evaluated through CST studio software and realized through a micro-forge CNC.

The probe was linked to a vector network analyzer MiniVNA-TINY [42] for measuring return loss in the chosen operating frequency range of 1.9–2.6 GHz, with a sampling frequency of 0.5 MHz. The VNA was calibrated using the calibration kit provided by the WIMO manufacturing company based on SMA open, short, and 50 Ω terminations.

Plexiglass support was realized ad hoc to guarantee the proper positioning of the Petri dishes over the probe. Moreover, four markers were drawn to ensure placement repeatability and to avoid misalignments between the dishes and the sensitive element during the tests.

### 2.3. Data Processing

The *RL* data were fitted using a Lorentzian curve (Equation (2)) through a non-linear least-squares method:

(2) $$L\left(x\right)=\frac{A_{p}}{1+{\left(\frac{x-x_{p}}{x_{u}-x_{l}}\right)}^{2}},\;$$

where *A_p_* is the peak amplitude, *x_p_* is the peak abscissa (frequency), and *x_l_* and *x_u_* represent the lower and upper bounds of the abscissa corresponding to half the amplitude of the distribution, respectively.

The fitting process allowed the determination of three key parameters: the minimum return loss (*minRL*), resonant frequency (*f_r_*), and full width at half maximum (*FWHM*). Outliers in the datasets were identified and eliminated using the ROUT method, applying a false discovery rate (*Q*) of 0.01% [43], which combines robust linear regression and outlier removal. For each parameter of each sample, the mean value ($\bar{m}$), standard deviation (*SD*), and coefficient of variation (*CV*) were calculated based on the ten repetitions. An *F* test on the sum-of-squares was performed to compare two different fit models—a straight line (*SL*, *y = nx + q*) and a horizontal line (*HL*, *y =* $\bar{m}$)—to assess the drift of the parameters of interest over time. There was approximately a 70 s interval between the first and tenth acquisition of the same sample, both for the pure culture medium and the biological samples.

To determine the feasibility of a faster but accurate measurement strategy for measuring adherent cells’ dielectric properties with just a single acquisition and to gain insights into its consistency and reliability, the absolute and the relative errors between the first measurement of each specimen and the average value of the ten measurements were computed:

(3) $$\mathrm{Error}\;\mathrm{of}\;\mathrm{First}\;\mathrm{Measurement}\;\;EFM=m_{1}-\bar{m},$$

(4) $$\;\;\mathrm{Relative}\;\mathrm{Error}\;\mathrm{of}\;\mathrm{First}\;\mathrm{Measurement}\;\;REFM\%=\left|\frac{EFM}{m}\right|\%,$$

where *EFM* is the distance between the first measurement *m*_1_ and the mean value $\bar{m}$ is computed over the ten measurements of each MUT replicate.

Data analysis was performed with GraphPad Prism 9.5.

## 3. Results and Discussion

By employing the ROUT method, we were able to detect and eliminate outliers from the dataset. Among the 1200 collected values, a total of 5 data points were identified as outliers and excluded from the subsequent descriptive statistical analysis.

**Figure 2:**
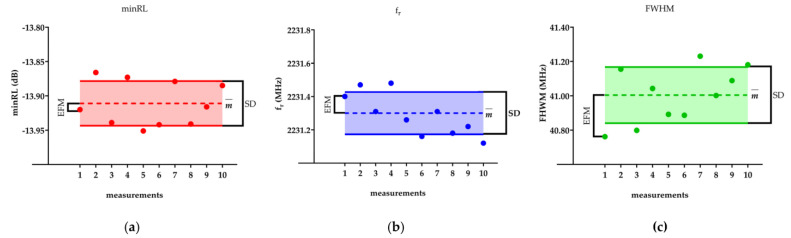
The figure reports for one single DMEM sample the mean value (m), the standard deviation (SD), and the error of the first measurement (*EFM*) for (**a**) *minRL*, (**b**) *fr*, and (**c**) *FHWM*. $\bar{m}$ is represented by the dashed line, while SD is the solid one.

To provide a visual representation of the statistical parameters, Figure 2 showcases an example of these parameters computed over the ten consecutive acquisitions for the minimum return loss (*minRL*), resonant frequency (*f_r_*), and full width at half maximum (*FWHM*) for one DMEM sample. The main statistical parameters in the figure are the mean value, standard deviation, and error of the first measurement (*EFM*).

**Figure 3:**
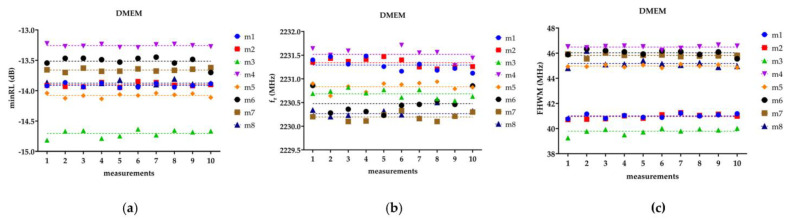
The figure reports the (**a**) *minRL*, (**b**) *f_r_*, and (**c**) *FHWM* for the ten repetitions of the eight DMEM samples. The dashed line represents the linear trend, or on the other hand, the average value.

Figure 3 depicts, as an example, the three fitting parameters (*minRL*, *f_r_*, *FWHM*) measured for each of the eight DMEM samples over ten repetitions. The figure shows that the ten acquisitions exhibit a scattered distribution around the average value for each of the measured parameters, displaying a random pattern without any discernible increasing or decreasing trend. The absence of a trend suggests that there is no systematic variation or bias in the measurements over time. The *F*-test between *SL* and *HL* highlights how the line resulting from the *SL* did not differ significantly from the *HL* for the three parameters of interest (*minRL*, *f_r_*, *FWHM)* and for all the tested DMEM samples. This finding suggests that there is no significant drift or variation in the parameters over time, indicating the stability and reliability of the measurement setup and methodology, thus reinforcing the validity of the measurement methodology and its suitability for investigating the dielectric properties of cells and biological tissues.

**Figure 4:**
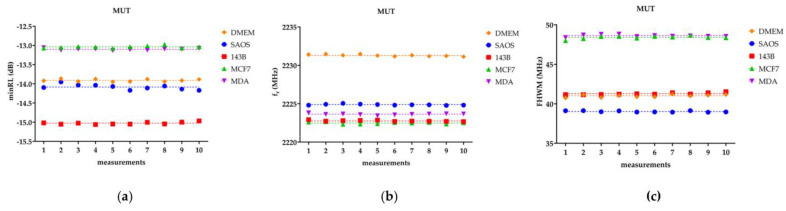
The dashed line represents the linear trend, or on the other hand, the average value. MDA stands for MDA-MB-231. (**a**) *minRL*, (**b**) *f_r_*, and (**c**) *FHWM* for the ten repetitions of one replicate of the five MUTs.

Figure 4 presents, as an example, the ten consecutive measurements obtained for one sample for each tested group: DMEM, SaOS-2, 143B, MCF-7, and MDA-MB-231. The measurements are displayed for the three fitting parameters (*minRL*, *f_r_*, *FWHM*). The linear trend line is also included for each replicate. 

Figure 4 points out that, for each sample, the ten repetitive measurements exhibit a constant linear trend. The presence of a constant linear trend, consistently observed across the tested groups, further supports the earlier findings that the parameters of interest (*minRL*, *fr*, *FWHM*) exhibit a stable behavior without any significant drift or variation over time. It is worth noting that the linear regression analysis, as mentioned previously, also confirmed the absence of significant differences between the *SL* and the *HL* fit for all the biological samples.

Table 1, Table 2 and Table 3 report the $\bar{m}$, SD, and CV values for the DMEM, the four tested cell lines, and *minRL*, *f_r,_* and *FWHM,* respectively.

Table 1 presents the mean $\bar{m}$, *SD*, and *CV* values for the *minRL* parameter of all the tested materials. For the DMEM samples, the mean values of *minRL* range from −13.26 dB to −14.7 dB across the eight samples, with small deviations, as indicated by the SD values ranging from 0.02 dB to 0.07 dB. The *CV* values are consistently low, ranging from 0.15% to 0.52%, indicating a good level of accuracy and reproducibility in the measurements.

Similar results in terms of variations were obtained for the SaOS-2 cell line, with average *minRL* values in the range of −13.22 dB to −15.57 dB and *SD* values in the range of 0.02 to 0.07 dB. The *CV* values were also in line with those of DMEM (from 0.20% to 0.50%), indicating a low variability in the *minRL* within this cell line.

The variability was even a little lower for the other three tested cell lines, namely 143B, MCF-7, and MDA-MB-231. Indeed, values of *minRL* ranged from −12.71 dB to −15.58 dB, from −12.73 dB to −13.92 dB, and from −12.51 dB to −14.11 dB, respectively, and the SD was always in the range 0.02 dB to 0.05 dB, with CV values in the range of 0.13% to 0.32%, indicating a very low variability in the measured parameter.

In general, the presence of biological materials does not significantly impact the measurement variability, and the low SD and CV values highlight the stability and consistency of the measurements.

Table 2 presents the $\bar{m}$, *SD,* and *CV* values for the *f_r_* parameter. For the DMEM samples, the mean value of *f_r_* ranges from 2230.19 MHz to 2231.52 MHz, with very small deviations, as indicated by the SD values ranging from 0.09 MHz to 0.22 MHz. The CV values are, therefore, consistently low (<0.010%), indicating a high level of accuracy and reproducibility in the measurements.

Interestingly, for the *minRL*, similar results in terms of variance are obtained for the measured *f_r_*. SaOS-2 cells, indeed, returned SD and CV values consistent with those of pure DMEM, where 143B, MCF-7, and MDA-MB-231 cell lines showed even lower variance, with SD values always lower than 0.10 MHz and CV lower than 0.004%, both of them being about half of that obtained for DMEM and SaOS-2.

Table 3 presents the $\bar{m}$, *SD,* and *CV* values for the *f_r_* parameter. Moving on to the SAOS cell line, the mean *FWHM* values range from 39.03 MHz to 49.28 MHz and the *SD* ranges from 0.09 MHz to 0.20 MHz, while the *CV* values range from 0.23% to 0.45%. These results suggest a relatively narrow distribution and low variability in the *FWHM* measurements for SAOS-2. Similarly, for the 143B cell line, the mean *FWHM* values range from 39.33 MHz to 48.91 MHz and the *SD* ranges from 0.086 MHz to 0.21 MHz, with corresponding *CV* values in the range of 0.18% to 0.43%. These findings indicate a consistent *FWHM* parameter across the different samples of 143B. For the MCF-7 cell line, the mean *FWHM* values range from 44.12 MHz to 49.54 MHz and *SD* values are in the range of 0.12 MHz to 0.20 MHz, while the *CV* values range from 0.26% to 0.45%. These results demonstrate a relatively stable *FWHM* parameter for MCF-7. Lastly, the MDA-MB-231 cell line exhibits mean *FWHM* values ranging from 45.42 MHz to 49.77 MHz and an *SD* in the range of 0.10 MHz to 0.21 MHz, with corresponding *CV* values ranging from 0.22% to 0.44%.

Finally, Table 4 shows the relative error of the first measurement for the *minRL*, *fr*, and *FWHM* of the five MUTs.

The *REFM*% values for all the parameters and all the tested groups are consistently lower than 1.5%. In detail, the *REFM*% values for the return loss and the resonance frequency are always lower than 0.800% and 0.018%, respectively, and are up to 1.4% only for the *FWHM*. These findings suggest that the measurement methodology that relies on a single acquisition of the return-loss signal introduces minimal error in quantifying the parameters of interest. In particular, the resonant frequency stands out as the most robust parameter for repetitive measurements. Of note, the resonance frequency was also demonstrated to be the most sensitive and robust parameter for discriminating between different cell lines and assessing their aggressiveness [28], and the results provided here further confirm its goodness in characterizing cell lines and evaluating their aggressiveness. However, the results in Table 4 emphasize the measurement procedure’s efficacy by demonstrating the general low error introduced in the determination of the main parameters. The low *REFM*% values indicate high precision and reproducibility in the measurements, which is crucial for reliable analysis and the discrimination of different cell lines.

The results reported in Table 1, Table 2 and Table 3 indicate minimal variability, demonstrating that the measurements are highly reproducible and comparable between DMEM and the tested cell lines. This finding substantiates the reliability and robustness of the measurement procedure and supports its suitability for accurately characterizing the dielectric properties of biological samples.

To further validate the accuracy of the measurements, a comparison was made between the mean value of the first measurement (*MVF*) and the mean values of the means (*MoMs*) or, on the other hand, the average of the ten repetitions for each of the eight replicates. The comparison revealed that the difference between the two values was consistently lower than 0.5% for all the tested materials. For example, when considering DMEM, the MVF for *minRL* was −13.84 ± 0.46 dB, while the *MoM* was −13.86 ± 0.43 dB. Similarly, the MVF for *fr* was 2230.92 ± 0.51 MHz and the *MoM* was 2230.83 ± 0.51 MHz. For *FWHM*, the MVF was 43.59 ± 2.86 MHz and the *MoM* was 43.78 ± 2.71 MHz. These small differences between the *MVF* and *MoM* values further support the consistency and accuracy of the measurement methodology. This outcome confirms that the first measurement provides representative data of the entire dataset, and the subsequent repetitions yield results that closely align with the overall mean values. Overall, the combined results from Table 1, Table 2, Table 3 and Table 4 underscore the precision, reliability, and robustness of the measurement methodology employed in the analysis of the dielectric properties of biological materials. The low scattering among consecutive measurements and the minimal differences between *MVF* and *MoM* values affirm the suitability of the procedure for the accurate characterization and discrimination of different cell lines based on their electrical properties.

In the literature, many studies focus on specific cell lines and employ different types of sensors and frequency ranges to measure the relevant parameters. In particular, [23] emphasized the measurement of complex permittivity (ε′ and ε″) in the frequency range of 0.2 GHz to 13.6 GHz. The procedure involves averaging multiple acquisitions and conducting three replicates for each specimen for MCF-10A, Hs578T, MDA-MB-231, MCF-7, T-47D, HT-29, and three types of culture medium. Moreover, Refs. [24,25] applied an open-ended coaxial probe to measure complex permittivity (ε*) and conductivity (σ) within the frequency range of 0.5 GHz to 10.0 GHz and 2.0 GHz to 50.0 GHz, focusing on hFOB and SaOS-2 cells in DMEM culture medium. Both studies utilize an average of multiple acquisitions to ensure accurate and representative measurements. In [28], the frequency range is 1.0 GHz to 3.0 GHz, and the parameters of interest include minimum reflection loss (*minRL* in dB), resonant frequency (*fr* in GHz), and full width at half maximum (*FWHM* in MHz). The study performs single acquisitions with eight replicates per specimen. Finally, in [26], authors adopt a similar approach by employing split-ring resonators to study SW620, Hs578T, and MCF-7 cells. This study analyzes the resonant frequency (*fr* in GHz), frequency shift (Δ*fr* in MHz), and Q-factor within the frequency range of 1.0 GHz to 8.0 GHz. Table 5 shows the procedure employed to test cells’ dielectric properties.

Due to the heterogeneity of methods and measured parameters found in the literature and the diversity of measurement protocols used (such as the type and volume of the medium, number of cells, and their concentration), a direct comparison of the absolute values of the parameters is not possible. However, since a standardized measurement protocol is not well established, we believe that our findings about the testing procedure could also be proposed for the other methods reported since they concern similar MUTs for similar dielectric properties.

In any case, considering the different cell numbers employed in the research studies (from 4 × 10^5^ to 12 × 10^5^), we conducted an extrapolation of the dielectric constant at the natural frequency of our sensor for the MCF-7, MDA-MB-231, and SaOS-2 cell lines based on data from the literature [24,25,27]. The results indicated that the SaOS-2 cell line exhibited a lower dielectric constant (*ε* = 74.5) than the other two lines, leading to a higher resonance frequency (1942 MHz). On the other hand, MDA-MB-231 exhibited a lower dielectric constant (*ε* = 75) than MCF-7 (*ε* = 75.5), resulting in a higher resonant frequency (1940 MHz against 1938 MHz). Even if obtained from an extrapolation, it is interesting to note that these findings are in agreement with the measurements obtained from our sensor, where SaOS-2 shows an average *f_r_* of 2225.06 ± 0.42 MHz, MCF-7 an average *f_r_* of 2222.4 ± 0.26, and MDA-MB-231 an average *f_r_* of 2223.71 ± 0.19.

## 4. Conclusions

In this work, we propose a comprehensive descriptive statistical analysis of a dataset comprising 400 return-loss measurements, for a total of 1200 data points, to establish an optimal measurement methodology for microwave-based sensors. Indeed, even if the measurement of dielectric properties has significantly impacted various scientific fields over the past two decades and microwave-based sensors have emerged as valuable tools in the biological and biomedical domains, offering non-invasive methods for early-stage prognosis of diseases, including malignancies, there is a lack of standardized testing procedures for evaluating cells’ and tissues’ dielectric properties.

Experimental results revealed consistent and stable behavior of the measured parameters (*minimum return loss*, *resonant frequency*, and *full width at half maximum*) across ten consecutive measurements of the same sample. The measurements exhibited a constant linear trend without any significant drift or variation, supporting the reliability and reproducibility of the measurement procedure. The analysis of the mean values, standard deviations, and coefficients of variation for the different cell lines demonstrated the stability and consistency of the measurements. Moreover, the low *REFM%* values (0.8% for *minRL*, 0.3% for *fr*, and 1.4% for *FWHM*) indicate high accuracy and reproducibility in the measurements, which is crucial for reliable analysis and the discrimination of different cell lines, suggesting that the single measurement provides representative data of the entire set of ten repetitive measurements, thus allowing a substantial reduction in time and, most of all, the preservation of the biological specimens, without compromising the measurement accuracy.

## Figures and Tables

**Table 1:** *minRL* for the five *MUTs*.

*minRL*		m_1_	m_2_	m_3_	m_4_	m_5_	m_6_	m_7_	m_8_
DMEM	$\bar{m}$ (MHz)	−13.91	−13.9	−14.7	−13.26	−14.08	−13.52	−13.66	−13.88
	*SD* (MHz)	0.03	0.03	0.06	0.02	0.03	0.07	0.03	0.04
	*CV* (%)	0.22%	0.22%	0.41%	0.15%	0.21%	0.52%	0.22%	0.29%
SaOS-2	$\bar{m}$ (MHz)	−14.08	−15	−15.57	−13.22	−13.6	−13.8	−13.39	−13.27
	*SD* (MHz)	0.07	0.03	0.06	0.04	0.03	0.03	0.02	0.08
	*CV* (%)	0.50%	0.20%	0.39%	0.30%	0.22%	0.22%	0.15%	0.60%
143B	$\bar{m}$ (MHz)	−15.03	−15.58	−14.28	−14.96	−13.44	−13.12	−12.71	−15.12
	*SD* (MHz)	0.03	0.05	0.03	0.02	0.03	0.03	0.03	0.03
	*CV* (%)	0.20%	0.32%	0.21%	0.13%	0.22%	0.23%	0.24%	0.20%
MCF-7	$\bar{m}$ (MHz)	−13.04	−12.73	−13.66	−13.57	−13.92	−12.88	−13.66	−13.89
	*SD* (MHz)	0.03	0.03	0.03	0.04	0.04	0.03	0.04	0.04
	*CV* (%)	0.23%	0.24%	0.22%	0.29%	0.29%	0.23%	0.29%	0.29%
MDA-MB-231	$\bar{m}$ (MHz)	−13.1	−12.51	−12.76	−13.33	−14.11	−14.1	−13.58	−13.54
	*SD* (MHz)	0.02	0.02	0.04	0.02	0.04	0.03	0.02	0.04
	*CV* (%)	−13.91	−13.9	−14.7	−13.26	−14.08	−13.52	−13.66	−13.88

**Table 2:** *f_r_* for the five *MUTs*.

*f_r_*		m_1_	m_2_	m_3_	m_4_	m_5_	m_6_	m_7_	m_8_
DMEM	$\bar{m}$ (MHz)	2231.29	2231.34	2230.69	2231.52	2230.83	2230.48	2230.19	2230.27
	*SD* (MHz)	0.13	0.09	0.09	0.12	0.10	0.22	0.09	0.11
	*CV* (%)	0.006%	0.004%	0.004%	0.005%	0.004%	0.010%	0.004%	0.005%
SaOS-2	$\bar{m}$ (MHz)	2224.88	2225.75	2225.14	2224.6	2225.19	2225.18	2224.34	2225.4
	*SD* (MHz)	0.08	0.04	0.07	0.10	0.10	0.20	0.09	0.07
	*CV* (%)	0.004%	0.002%	0.003%	0.004%	0.004%	0.009%	0.004%	0.003%
143B	$\bar{m}$ (MHz)	2222.76	2222.81	2222.86	2223.64	2222.87	2223.49	2223.59	2223.26
	*SD* (MHz)	0.09	0.05	0.07	0.10	0.09	0.10	0.06	0.09
	*CV* (%)	0.004%	0.002%	0.003%	0.004%	0.004%	0.004%	0.003%	0.004%
MCF-7	$\bar{m}$ (MHz)	2222.48	2222.2	2222.44	2222.23	2222.64	2222.17	2222.21	2222.83
	*SD* (MHz)	0.10	0.08	0.06	0.08	0.08	0.07	0.05	0.07
	*CV* (%)	0.004%	0.004%	0.003%	0.004%	0.004%	0.003%	0.002%	0.003%
MDA-MB-231	$\bar{m}$ (MHz)	2223.63	2223.62	2223.67	2223.85	2223.94	2223.31	2223.78	2223.89
	*SD* (MHz)	0.10	0.08	0.06	0.08	0.09	0.08	0.07	0.08
	*CV* (%)	0.004%	0.004%	0.003%	0.004%	0.004%	0.004%	0.003%	0.004%

**Table 3:** *FWMH* for the five *MUTs*.

*FWHM*		m_1_	m_2_	m_3_	m_4_	m_5_	m_6_	m_7_	m_8_
DMEM	$\bar{m}$ (MHz)	41.00	40.96	39.79	46.49	44.97	46.03	45.81	45.18
	*SD* (MHz)	0.16	0.19	0.24	0.12	0.1	0.22	0.12	0.39
	*CV* (%)	0.39%	0.46%	0.60%	0.26%	0.22%	0.48%	0.26%	0.86%
SaOS-2	$\bar{m}$ (MHz)	39.03	40.47	39.80	48.90	49.28	48.27	48.95	46.41
	*SD* (MHz)	0.09	0.15	0.18	0.15	0.16	0.11	0.17	0.2
	*CV* (%)	0.23%	0.37%	0.45%	0.31%	0.32%	0.23%	0.35%	0.43%
143B	$\bar{m}$ (MHz)	41.28	39.59	42.02	48.91	47.55	47.7	48.5	39.33
	*SD* (MHz)	0.13	0.16	0.13	0.15	0.14	0.086	0.21	0.15
	*CV* (%)	0.31%	0.40%	0.31%	0.31%	0.29%	0.18%	0.43%	0.38%
MCF-7	$\bar{m}$ (MHz)	48.41	49.54	46.78	46.76	45.95	48.5	46.78	44.12
	*SD* (MHz)	0.19	0.15	0.12	0.16	0.12	0.16	0.15	0.2
	*CV* (%)	0.39%	0.30%	0.26%	0.34%	0.26%	0.33%	0.32%	0.45%
MDA-MB-231	$\bar{m}$ (MHz)	48.61	49.77	48.92	48.43	45.89	45.71	46.39	45.42
	*SD* (MHz)	0.14	0.023	0.21	0.17	0.11	0.10	0.12	0.20
	*CV* (%)	0.29%	0.05%	0.43%	0.35%	0.24%	0.22%	0.26%	0.44%

**Table 4:** *REFM*% for the five *MUTs*.

*REFM*%		m_1_	m_2_	m_3_	m_4_	m_5_	m_6_	m_7_	m_8_
DMEM	*minRL*	41.00	40.96	39.79	46.49	44.97	46.03	45.81	45.18
	*fr*	0.16	0.19	0.24	0.12	0.1	0.22	0.12	0.39
	*FWHM*	0.39%	0.46%	0.60%	0.26%	0.22%	0.48%	0.26%	0.86%
SaOS-2	*minRL*	39.03	40.47	39.80	48.90	49.28	48.27	48.95	46.41
	*fr*	0.09	0.15	0.18	0.15	0.16	0.11	0.17	0.2
	*FWHM*	0.23%	0.37%	0.45%	0.31%	0.32%	0.23%	0.35%	0.43%
143B	*minRL*	41.28	39.59	42.02	48.91	47.55	47.7	48.5	39.33
	*fr*	0.13	0.16	0.13	0.15	0.14	0.086	0.21	0.15
	*FWHM*	0.31%	0.40%	0.31%	0.31%	0.29%	0.18%	0.43%	0.38%
MCF-7	*minRL*	48.41	49.54	46.78	46.76	45.95	48.5	46.78	44.12
	*fr*	0.19	0.15	0.12	0.16	0.12	0.16	0.15	0.2
	*FWHM*	0.39%	0.30%	0.26%	0.34%	0.26%	0.33%	0.32%	0.45%
MDA-MB-231	*minRL*	48.61	49.77	48.92	48.43	45.89	45.71	46.39	45.42
	*fr*	0.14	0.023	0.21	0.17	0.11	0.10	0.12	0.20
	*FWHM*	0.29%	0.05%	0.43%	0.35%	0.24%	0.22%	0.26%	0.44%

**Table 5:** Overview of the measurement procedure for breast cancer and osteosarcoma cells’ detection.

Ref.	Kind of Cells	Kind of Sensor	Freq. Range [GHz]	Measured Parameters ^1^	Procedure
[24]	MCF-10A, Hs578T, MDA-MB-231, MCF-7, T-47D, HT-29, culture medium (three types)	Open-ended coaxial probe	0.2–13.6	*ε*′, *ε*″	Average of multiple acquisitions. Three replicates for specimens
[25]	hFOB, SaOS-2, DMEM	Open-ended coaxial probe	0.5–10.0	ε*, *σ*	Single acquisition on two replicates
[26]	hFOB, SaOS-2, C2C12	Open-ended coaxial probe	2.0–50.0	*ε*′, *ε*″	Average of multiple acquisitions.
[27]	SW620, Hs578T, MCF-7	Split-ring resonators	1.0–8.0	*f_r_* (GHz), Δ*f_r_* (MHz), *Q-factor*	Simulation
[29]	DMEM, SaOS-2, 143B, MCF-7, MDA-MB-231	Circular-patch resonator	1.0–3.0	*minRL* (dB), *f_r_* (GHz), *FWHM* (MHz)	Single acquisition. Eight replicates for specimens

^1^ ε′, ε″, ε* are respectively real, imaginary and complex permittivity σ is the conductivity.

## Data Availability

Not applicable.
